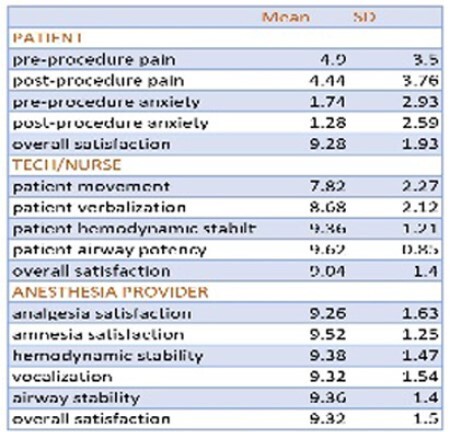# 725 Satisfaction of Patient, Burn Nurse/Tech and Anesthesia Provider for Monitored Anesthesia Care Burn Dressing Change

**DOI:** 10.1093/jbcr/irae036.268

**Published:** 2024-04-17

**Authors:** Fatma O Ulusan Sayali, Anthony Kovac, Melinda Becker, Dhaval Bhavsar, Duncan Nickerson, Katherine Golson, Niaman Nazir

**Affiliations:** University of Kansas Medical Center, Kansas City, KS; University of Kansas Medical Center, Roeland Park, KS; The University of Kansas Health System, Kansas City, KS; University of Kansas Health System, Kansas City, KS; University of Kansas Medical Center, Kansas City, KS; University of Kansas Medical Center, Roeland Park, KS; The University of Kansas Health System, Kansas City, KS; University of Kansas Health System, Kansas City, KS; University of Kansas Medical Center, Kansas City, KS; University of Kansas Medical Center, Roeland Park, KS; The University of Kansas Health System, Kansas City, KS; University of Kansas Health System, Kansas City, KS; University of Kansas Medical Center, Kansas City, KS; University of Kansas Medical Center, Roeland Park, KS; The University of Kansas Health System, Kansas City, KS; University of Kansas Health System, Kansas City, KS; University of Kansas Medical Center, Kansas City, KS; University of Kansas Medical Center, Roeland Park, KS; The University of Kansas Health System, Kansas City, KS; University of Kansas Health System, Kansas City, KS; University of Kansas Medical Center, Kansas City, KS; University of Kansas Medical Center, Roeland Park, KS; The University of Kansas Health System, Kansas City, KS; University of Kansas Health System, Kansas City, KS; University of Kansas Medical Center, Kansas City, KS; University of Kansas Medical Center, Roeland Park, KS; The University of Kansas Health System, Kansas City, KS; University of Kansas Health System, Kansas City, KS

## Abstract

**Introduction:**

Monitored Anesthesia Care (MAC) is moderate to deep anesthesia useful for painful procedures such as burn dressing change. Ratings by patient and nurse of pain and anxiety during burn wound care has been previously described. Concurrent satisfaction ratings of analgesia and sedation by the patient, burn nurse/tech and anesthesia provider has not been previously reported. Purpose: to evaluate satisfaction of patient, burn nurse/tech and anesthesia provider during MAC amnesia/analgesia for burn dressing change.

**Methods:**

This prospective observational study was approved by our hospital Human Subjects Committee. Inclusion criteria were adult in-patients older than 18 years receiving dressing change requiring moderate to deep analgesia and sedation. Informed consent was obtained and data collected from January 1, 2022, to September 1, 2023. Following dressing change, satisfaction on 10-point scale (0 = least satisfied; 10 = most satisfied) was evaluated among patients, burn nurse/techs, and anesthesia providers. Only the first dressing change was evaluated. Patient demographics, co-morbidities, duration of dressing change/procedure and burn injury effects such as etiology, % TBSA, burn degree was studied to determine whether these variables influenced satisfaction and/or efficacy. T-TEST and ANOVA were used to evaluate data. A p< 0.05 value was set as statistical significance.

**Results:**

50 inpatient dressing changes were evaluated. Regarding demographic data, variables with greatest % were Caucasian males (72%), ASA physical class 3 (66%), flame etiology (50%), smoking history (28%), and substance abuse history (40%). Patient, burn tech/nurse and anesthesia provider satisfaction data rated on a 0 (least) to 10 (most) scale are listed in the Table. Overall satisfaction (mean +/- SD) among patients was 9.28 +/- 1.93; burn tech/nurse 9.04 +/- 1.4 and anesthesia providers 9.32+/- 1.5 with no significant difference between groups. There was no correlation of patient weight, BMI, ASA physical class, %TBSA, burn degree or length of procedure (dressing change) to patient, burn tech/nurse or anesthesia provider satisfaction.

**Conclusions:**

MAC anesthesia was overall felt to be a satisfactory technique that can effectively be used for burn-dressing change. Anesthesia providers were satisfied with their MAC technique. All episodes of apnea and hypotension that occurred were properly identified and correctly treated without ill effects. Burn nurse/techs were satisfied that the amount of MAC administered allowing the occurrence of minimal or rare patient movement, limb resistance or verbalization. Patients were satisfied regarding the amount of their analgesia and amnesia, and agreeable to receive MAC for future dressing changes.

**Applicability of Research to Practice:**

MAC anesthesia can be considered a safe and effective anesthesia technique with overall good concurrent satisfaction of the patient, burn nurse/tech and anesthesia provider.